# Assessment of Multiple Sclerosis Patients’ Knowledge and Behavioral Practice Regarding COVID-19 in Saudi Arabia

**DOI:** 10.7759/cureus.32781

**Published:** 2022-12-21

**Authors:** Nimah Alsomali, Khaled A Amer, Arwa A Almutairi, Razan M Almasoudi, Sarah W Alkhonizy, Halimah H Faqih, Hajar A Alkhamis, Hanadi M AlGarni

**Affiliations:** 1 Neuroscience, King Fahad Medical City, Riyadh, SAU; 2 College of Medicine, King Khalid University, Abha, SAU; 3 College of Medicine, Taif University, Taif, SAU; 4 College of Medicine, Ibn Sina National College for Medical Studies, Jeddah, SAU; 5 Applied Medical Sciences, King Abdulaziz University, Jeddah, SAU; 6 College of Medicine, Alfaisal University, Riyadh, SAU; 7 College of Medical Rehabilitation, Qassim University, Qassim, SAU; 8 College of Science, Imam Abdulrahman Bin Faisal University, Dammam, SAU

**Keywords:** saudi arabia, attitude, knowledge, pandemic, covid-19, multiple sclerosis

## Abstract

Background

Coronavirus disease 2019 (COVID-19) is caused by a coronavirus subtype called severe acute respiratory syndrome coronavirus 2 (SARS-CoV-2). It is crucial to control the spread of coronavirus by understanding the disease and practicing the measures adopted during the COVID-19 pandemic. Multiple sclerosis (MS) is a central nervous system immune-mediated inflammatory demyelinating disease. COVID-19 infection may exacerbate the MS disease and its relapses. Therefore, MS patients are more susceptible to infection because of their immunosuppressive or immunomodulatory medications.

Objective

We aimed to evaluate the knowledge, attitude, and behaviors of patients with MS in Saudi Arabia regarding the COVID-19 pandemic.

Method

A quantitative observational cross-sectional study was conducted. MS patients in Saudi Arabia were included in the study population. Data were collected via an online self-reported questionnaire from 214 participants from November 2020 to June 2021.

Results

A total of 214 MS patients participated in this study. The gender distribution showed that the male participants represented 38.3% (n = 82), while female participants accounted for 61.7% (n = 132). Most MS patients understood the COVID-19 preventive measures. The mean knowledge score was 15.7 (SD = 2.34, range: 1-20), showing an appropriate level of knowledge. While the mean behaviors score was 6.1 (SD = 1.2, range: 2-9), showing good behaviors. The mean score for attitude was 5.4 (SD = 1.77, range: 1-8), showing optimistic attitudes. However, a closer analysis of the participants’ answers showed that 74.3% of the patients agreed that the treatment plan should be discussed with their doctors during the pandemic. In addition, almost half of the participants (49.1%) agreed that being an MS patient means they are at higher risk of getting infected by the virus. Data also showed that 17% of patients continued to attend social events involving many people. Also, 28.0% of the patients reported being in crowded places.

Conclusion

MS patients’ risk of COVID-19 might be linked to their knowledge, attitude, and behaviors. Our results suggest that although MS patients have a high knowledge and good attitude and behaviors, it is still essential to have health education programs among MS patients to reduce the risk of SARS-CoV-2 infection and the impact of the COVID-19 pandemic on MS patients’ care. Knowledge, attitude, and behaviors toward the COVID-19 pandemic can highlight the importance of education programs and translate the findings into action to minimize the disease risk.

## Introduction

Coronavirus disease 2019 (COVID-19) is an infectious disease caused by a coronavirus subtype called severe acute respiratory syndrome coronavirus 2 (SARS-CoV-2). SARS-CoV-2 is a ribonucleic acid (RNA) virus that may lead to mild disease (fever, dry cough, and shortness of breath) or severe, critical disease (respiratory failure, acute respiratory distress syndrome [ARDS], and multiple organ dysfunction syndromes [MODS]) [1,2].

Late in December 2019, the first case infected with pneumonia without knowing the exact etiology was announced in Wuhan, central China’s Hubei province [3]. On January 30, 2020, the World Health Organization (WHO) declared the COVID-19 outbreak a pandemic, urging governments to take immediate and aggressive measures to combat COVID-19 spreading further [4]. Saudi Arabia has gone above and beyond to ensure the safety of its citizens by putting laws in place and limiting the spread of the virus. Also, the Saudi government has supported its citizens economically by ensuring the unemployment rate stays low, giving financial support to those who need it, and ensuring there is no inflation on the price of necessary products to the consumers [5]. The ministry of health (MOH) played a huge part in this pandemic; about 64,234 Saudi physicians, 139,798 Saudi nurses, 8,273 Saudi pharmacists, and 75,000 Saudi health support services employees risked their lives to combat the virus. Moreover, 494 hospitals in the Kingdom and 75,225 hospital beds were provided to the community [6].

The Saudi MOH was transparent in providing information about the virus to the public. It spread awareness regarding COVID-19 through social media, precautionary messages, and combating rumors and false information about the disease; it also held a daily conference updating its citizens on the new developments of COVID-19 and vaccines [7]. About 19.6M have received the first dose of the COVID-19 vaccine, and about 2.2M are fully vaccinated, which equals 6.4% of the population [8]. In Saudi Arabia, there is a family-based culture, and with this pandemic, people have been social distancing. Thus, some stopped visiting their families to take precautions, including wearing masks everywhere and limiting family gatherings.

Multiple Sclerosis (MS) is a central nervous system immune-mediated inflammatory demyelinating disease that affects more than 2 million people worldwide and is a leading cause of severe neurological disability in adults [9]. According to recent studies, MS has a moderate-to-high prevalence throughout the Middle East and North Africa [10]. Patients with MS require long-term disease-modifying therapy (DMT) to help regulate their disease progression and reduce relapse rates; DMT may impact the risk profile of MS patients, while interferon beta therapy is associated with a lower risk of infection [11,12].

Relapsing-remitting multiple sclerosis (RRMS) is the most common variety of MS; around 85% of persons have it. RRMS is characterized by the emergence of acute or subacute neurological symptoms from which patients may recover entirely or partially. Relapses may occur at irregular periods after that. Patients may develop neurological deficits and disabilities if their recovery from relapses is insufficient. A recent study has shown that relapses impact the development of residual deficits, with up to 42% of patients seeing a quantifiable and consistent increase in impairment at follow-up after relapse [13].

Secondary progressive multiple sclerosis (SPMS) is a type of MS that may develop into a disease pattern of slow progression with irreversible neurological deficiency and disability over time. The proportion of persons who get progressive disease rises with the time they are followed. According to a Canadian study, 41% of persons with RRMS entered the secondary progressive phase within 6-10 years of beginning, rising to 58% between 11 and 15 years [14]. Eight people with SPMS may experience relapses that are overlaid. On the other hand, primary progressive multiple sclerosis (PPMS) is a type of progressive MS that is characterized by progressive disease from the start, with a slow accumulation of neurological deficiency or disability and no relapse or remission, and accounts for around 10-15% of MS cases [15]. The average age of onset, around 40 years, is later than in RRMS, and males are affected in more significant numbers than women, resulting in a nearly equal male: female ratio of [15].

The disease onset age and development pathway are comparable to SPMS’ progressive phase [16]. PRMS is characterized as a disease that progresses over time with relapses. The disease’s progressive nature predominates, and PRMS is thought to be quite similar to PPMS; superimposed relapses affect around 10-15% of persons with PPMS [17,18]. COVID-19 may cause MS patients stress, including anxiety, depression, and panic attacks, and increase respiratory diseases, such as severe pneumonia and pulmonary edema, which may develop into ARDS [19,20]. COVID-19 is a viral infection that may exacerbate the disease and its relapses. There is a need for more studies to establish the relationship between the severities and risk of immunity [21]. Patients who use immunosuppressive or immunomodulatory medication are more prone to infections [22].

Despite enormous national efforts to control the epidemic, reducing the rising number of COVID-19 cases requires adherence to the advised preventive measures. These measures are affected mainly by public behaviors and knowledge [23]. A recent study in Iran was done to assess the attitude and knowledge of MS patients in response to COVID-19, revealing that MS patients had a strong understanding of COVID-19 preventive measures and transmission routes. They did, however, get less information from their doctors on medication usage control, admission centers, and physician consultation in the event of COVID-19 infection [23]. Individuals’ awareness of COVID-19 is linked to their attitude toward the condition and degree of stress. Furthermore, COVID-19 disinformation and unfavorable attitudes increase the likelihood of infection and act as a roadblock to COVID-19 control [24]. It is essential to assess MS patients’ knowledge, attitudes, and various behavioral patterns linked to COVID-19 to reduce the risk of SARS-CoV-2 infection and the impact of the COVID-19 pandemic on MS patients’ care [25].

It is crucial to control the spread of coronavirus by understanding the disease and practicing the measures adopted during the COVID-19 pandemic. COVID-19 may exacerbate MS disease and its relapses. Therefore, MS patients might be more susceptible to the infection, hence, we aim in this study to investigate the knowledge, attitudes, and practices of multiple sclerosis patients toward the COVID-19 pandemic.

## Materials and methods

A quantitative observational cross-sectional study was conducted at the National Neuroscience Institute, King Fahad Medical City (KFMC), Riyadh, Saudi Arabia. With the aid of patient support groups, patients with confirmed MS in Saudi Arabia were invited to take part in the study. Ethical approval was granted before data collection from the Second Health Cluster Institutional Review Board (IRB) with IRB Log Number (20-653). The questionnaire was sent to MS patients in Saudi Arabia from November 2020 to June 2021.

Questionnaire

An anonymous, self-administered online questionnaire was completed using Google Forms. The neurologists developed the questionnaire based on other studies that measured knowledge, attitudes, and perceptions toward MS. The questionnaire contained four sections: demographic information, knowledge, attitudes, and practice. The demographic section included gender, age, marital status, occupation, level of education, nationality, region, monthly income, time of diagnosis, type of MS, and type of medications. The knowledge section included 21 items that evaluated different aspects of COVID-19 knowledge. Each item had one correct or incorrect answer and an “I do not know” option. The attitude section contained eight items: agreeing, disagreeing, and a “fair” option. Finally, the practice section contains nine items, including yes and no answers, plus an “I do not know” option.

Participants

We calculated a required sample size of 214 using the formula (n = z2pq\d2), where Z is 1.96, and our desired accuracy is 0.05. The eligibility criteria required MS patients residing in Saudi Arabia, diagnosed with MS for ≥1 year, and aged ≥18 years. We excluded any patients who had unconfirmed diagnoses or suspected cases. The questionnaire link was electronically sent to social media sites. Data were collected in June 2021. The participants provided informed consent by clicking on the “agree to participate” icon on the first page of the electronic survey.

Data analysis plan

Statistical Package of Social Science software (SPSS 23 for mac) was used for analysis. Categorical variables were analyzed using chi-square χ2 tests and t-test for continuous variables; P<0.05 was considered statistically significant.

## Results

Demographic characteristics

**Figure 1:**
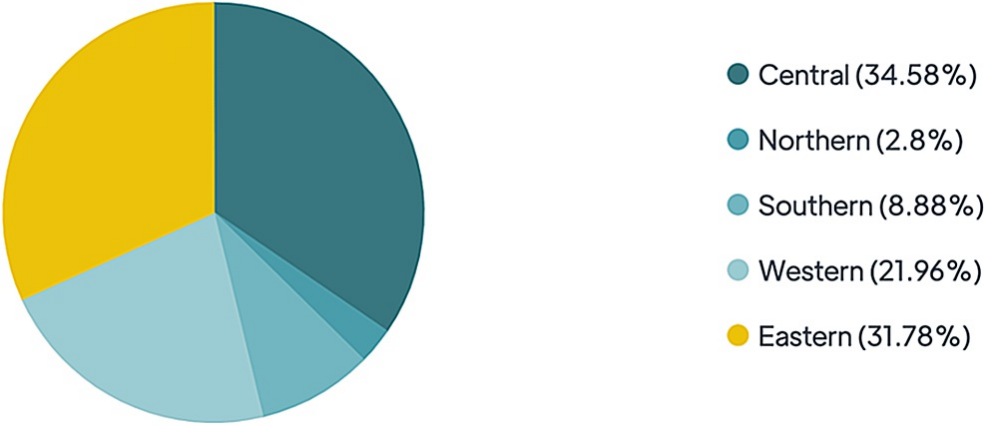
Regional distribution of the participants.

A total of 214 MS patients participated in this study. Table 1 shows that the male participants represented 38.3% (n = 82), while the female one was 61.7% (n = 132). The age of the studied group ranged from 18 to 64 years old; patients in the 25-34 age group represented 41.6% (n=89). Of all participants, 54.2% were single, while 45.8% were married. More than half (58.4%) of the respondents were unemployed. Most participants had a bachelor’s degree (36%, n=135), while the proportion with high school level education was 25.7% (n=55). Most participants were Saudis (92.5%); 31.8% of the whole sample was from the eastern region, 34.6% was from the central region, and 22% was from the western region (Figure 1). Regarding the monthly income, 39.7% of the participants had a low income (less than 3,000 Saudi Riyals) and 38.3% earned 3,000-10,000 Saudi Riyals income. Most patients included in the study (67, 31.3%) were diagnosed with MS more than 10 years ago, 62 (29.0%) of the patients were diagnosed 2 to five years ago, 49 (22.9%) were diagnosed with six to nine years ago, and around 36 (16.8%) were diagnosed one year ago or less. Eighty-eight (41.1%) of the study population had a relapsing-remitting form of the disease, and 35 (16.4%) had the progressive (primary and secondary) type. Unfortunately, 91 (42.5%) answered “I do not know” when asked about their MS type.

**Table 1:** Demographic variables of the study subjects.

	Frequency	Percent	Valid Percent	Cumulative Percent
Gender				
Male	82	38.3	38.3	38.3
Female	132	61.7	61.7	100.0
Age group				
35-44	68	31.8	31.8	88.8
45-54	19	8.9	8.9	97.7
55-64	5	2.3	2.3	100.0
Marital status				
Single	116	54.2	54.2	54.2
Married	98	45.8	45.8	100.0
Employment and educational level				
Employed	89	41.6	41.6	41.6
Unemployed	125	58.4	58.4	100.0
Less than high school	10	4.7	4.7	4.7
High school	55	25.7	25.7	30.4
Bachelor’s degree	135	63.1	63.1	93.5
Master’s degree	13	6.1	6.1	99.5
Doctoral degree	1	.5	.5	100.0
Nationality				
Saudi	198	92.5	92.5	92.5
Non-Saudi	16	7.5	7.5	100.0
Region				
Eastern	68	31.8	31.8	31.8
Western	47	22.0	22.0	53.7
Southern	19	8.9	8.9	62.6
Northern	6	2.8	2.8	65.4
Central	74	34.6	34.6	100.0
Income				
Less than 3,000	85	39.7	39.7	39.7
3000 to 10,000	82	38.3	38.3	78.0
10,000 to 20,000	36	16.8	16.8	94.9
More than 20,000	11	5.1	5.1	100.0
Total	214	100.0	100.0	100.0

When asked about the type of medication, the majority (n=71, 33.1%) answered “I do not know” the type of medication used, while 46 (21.5%) used IFN (Interferon), 22 (10.3%) used Tysabri, and 19 (8.9%) used Rituximab (Table 2).

**Table 2:** Types of medication used by the participants.

	Frequency	Percent	Valid Percent	Cumulative Percent
IFN (Interferon)	46	21.5	21.5	21.5
Glatiramer acetate (GA)	3	1.4	1.4	22.9
Fingolimod	18	8.4	8.4	31.3
Triflunomid	8	3.7	3.7	35.0
Tysabri	22	10.3	10.3	45.3
Rituximab	19	8.9	8.9	54.2
Dimethyl fumarate (DMF)	7	3.3	3.3	57.5
Ocrelizumab	17	7.9	7.9	65.4
No medication at the time	3	1.4	1.4	66.8
I don’t know	71	33.2	33.2	100.0
Total	214	100.0	100.0	100.0

Knowledge, attitude, and practice (KAP) scores

Knowledge, attitude, and practice (KAP) scores show that most MS patients understood the COVID-19 disease and preventive measures. The mean knowledge score was 15.7 (SD = 2.34, range: 1-20), showing an appropriate level of knowledge. While the mean score for practices was 6.1 (SD = 1.2, range: 2-9), showing good practices. The mean score for attitude was 5.4 (SD = 1.77, range: 1-8), showing optimistic attitudes (Table 3).

**Table 3:** The number of questions, range, scores and levels of knowledge, attitude, and practice among participants. SD: standard deviation.

Variables	Number of questions	Range of score	Total score (mean ± SD)
Knowledge	21	1-20	15.7850 ± 2.43614
Attitude	9	2-9	6.1308 ± 1.20316
Practice	8	1-8	5.4533 ± 1.77229

Assessment of knowledge

Regarding knowledge, around 60.3% of participants believed that SARS-CoV-2 could spread from person to person within close distance, 93.9% of the respondents knew that SARS-CoV-2 could be transmitted through cough and sneeze, 92.5% of the respondents believed that SARS-CoV-2 could be transmitted by touching a surface or object on which the virus is attached, and 64.5% of the respondent believed that animals could not cause COVID-19 to people. While 75.2% of the respondent knew that SARS-CoV-2 could be transmitted when a fever is not present, 93.9% of the respondent knew the main clinical symptoms of COVID-19, and 57.0% of the respondent agreed that the symptoms of the common cold are less common in infected people with COVID-19.

Additionally, 81.3% of participants answered that early symptomatic treatment could help most patients recover from the disease, but there is no effective cure for COVID-19. Also, 95.8% were aware that patients with severe chronic illnesses, such as heart or lung disease and diabetes, are at increased risk of developing life-threatening complications of COVID-19. Results also showed that 72% of the participants in the study believed that older adults with chronic illnesses tend to get severe symptoms of COVID-19, and 48.6% of the respondent believed that children do not appear to be at higher risk for COVID-19 than adults. Also, 93.5% believed that children or young people do not need to take precautionary measures to prevent SARS-CoV-2 transmission.

We found that 93.0% of patients believed that it is a must to wash their hands with soap and water or use a hand sanitizer containing at least 60% alcohol. About 75.7% of the participants believed that healthy food and drinking water increased the body’s immunity and resistance to COVID-19. While 98.1% believed that isolation and treatment of people infected with SARS-CoV-2 are effective ways to reduce the spread of the virus, and 95.3% of participants believed that someone in contact with someone infected with SAS-CoV-2 should be immediately quarantined, in an appropriate location, for a general observation period of 14 days. Finally, 97.7% of participants believed that to prevent transmission of SARS-CoV-2, people must also avoid going to crowded places and taking public transport. Table 4 revealed the association between demographic variables and knowledge, around 79% of females showed a higher proportion than males to agree that the common cold symptoms are less common in infected people with COVID-19 (p<0.002).

**Table 4:** Association between demographic variables and knowledge. SARS-CoV-2: severe acute respiratory syndrome coronavirus 2.

Unlike the common cold, congestion, runny nose, and sneezing are less common in people infected with SARS-CoV-2					
Gender	True	False	I don’t know	Total	Asymp. Sig. (2-sided)
Male	43	18	21	82	.002
Female	79	8	45	132	.003
Total	122	26	66	214	.933
After being in a public place, after nose-blowing, coughing, or sneezing, people must wash their hands with soap and water or use a hand sanitizer containing at least 60% alcohol for at least 20 seconds					
Gender	True	False	I don’t know	Total	Asymp. Sig. (2-sided)
Male	71	6	5	82	.005
Female	128	4	0	132	.002
Total	199	10	5	214	.001
People infected with SARS-CoV-2 cannot transmit the virus to others when a fever is not present					
Age	True	False	I don’t know	Total	Asymp. Sig. (2-sided)
18-24	2	25	6	33	
25-34	3	71	15	89	.043
35-44	1	51	16	68	.162
45-54	4	10	5	19	.707
55-64	0	4	1	5	
Total	10	161	43	214	
People should only wear a mask if they are infected with the virus or if they are caring for someone with a suspected SARS-CoV-2 infection					
Age	True	False	I don’t know	Total	Asymp. Sig. (2-sided)
18-24	1	32	0	33	
25-34	4	79	6	89	.049
35-44	8	60	0	68	.021
45-54	0	19	0	19	.106
55-64	1	4	0	5	
Total	14	194	6	214	

Moreover, 86% of males and 96.9% of females answered that washing hands prevents the spread of COVID-19 (p<0.005). Also, around 71% of the 25-34 age group believe that those infected with SARA-Cov-2 cannot transmit the virus to others when a fever is not present (p<0.043). However, 79% of the 25-34 age group believed they should not wear a mask if they are infected with the virus or care for someone with suspected SARS-CoV-2 infection (p<0.049).

Assessment of attitude 

The results showed that 35% of MS patients felt anxious whenever COVID-19 came to their minds, while the rest were neutral or disagreed. Around 68.7% of the patients thought that the pandemic and the virus would be successfully controlled. Almost half (49.1%) of the participants agreed that being an MS patient means they are at higher risk of getting infected by the virus. In addition, a significant number agreed that the strict measures that Saudi Arabia is following could help stop COVID-19 which equals 86%, and 66.8% thought to have an influential role in preventing the spread of the virus. Also, 87.9% strictly followed the quarantine guidelines, and 77.6% agreed that staying home helps reduce the spread of the virus. Lastly, 74.3% of the patients agreed that the treatment plan should be discussed with their doctors during the pandemic.

Association between demographic variables and attitude

When we talk about the association between demographic variables and attitude, a higher percentage of females agreed that being an MS patient means they are at higher risk of getting infected by the virus compared to males (p<0.026). Around 66.8% thought they had an effective role in preventing the spread of COVID-19, and most of them were between 25 and 44 years old (p<0.014). Seventy-three respondents were married, and 70 were not married (<0.0.23). Participants were asked to respond to the statement “I think that I have an effective role in preventing the spread of the virus” (Table 5).

**Table 5:** Association between demographic variables and attitude.

I think that I have an effective role in preventing the spread of the virus				
Age	Agree	Disagree	Neutral	Total
18-24	18	3	12	33
25-34	50	10	29	89
35-44	57	2	9	68
45-54	15	2	2	19
55-64	3	0	2	5
Total	143	17	54	214

Assessment of practice

Regarding the practice of MS patients towards COVID-19, when asked if washing hands with soap can reduce the spread of the virus, 97.7% agreed with the statement. On the other hand, a minority of the patients, 21.5%, agreed that avoiding touching the eyes, nose, and mouth can reduce the spread of the virus. In addition to that, 17.3% have recently been to a social event involving many people, while 28.0% of participants have been in a crowded place. Most (76.6%) avoided cultural behaviors such as shaking hands, and 86.9% of the patients practiced social distancing. Furthermore, 95.8% agreed that wearing masks during sickness reduces the spread of the virus, and 92.1% agreed that wearing masks in public places can reduce the spread of the virus. Lastly, 97.7% of the patients agreed that healthy people wearing masks could reduce the spread of the virus.

Association between demographic variables and practice

We found that, among MS patients, a significantly higher percentage of females than males reported not attending social events involving many people (p<0.030).

## Discussion

COVID-19 is caused by a coronavirus subtype called SARS-CoV-2. It is crucial to control the spread of coronavirus by understanding the disease and practicing the measures adopted during the COVID-19 pandemic. COVID-19 is a viral infection that may exacerbate MS disease and its relapses. Therefore, MS patients might be more susceptible to infection because of their immunosuppressive or immunomodulatory medications.

This study investigates the knowledge, attitudes, and practices of multiple sclerosis patients toward the COVID-19 pandemic. The findings could aid in modifying future efforts to best reach the groups with low levels of awareness. Data also showed a more significant response from the central region of Saudi Arabia due to the region’s dense population and the social media influencers who assisted in distributing the questionnaire. Most MS patients understood the COVID-19 disease and preventive measures. The mean knowledge score was 15.7 (SD = 2.34, range: 1-20), showing an appropriate level of knowledge. While the mean score for practices was 6.1 (SD = 1.2, range: 2-9), showing good practices. The mean score for attitude was 5.4 (SD = 1.77, range: 1-8), showing optimistic attitudes. However, a closer analysis of the participants’ answers showed that 74.3% of the patients agreed that the treatment plan should be discussed with their doctors during the pandemic. In addition, 49.1%, almost half of the participants, agreed that being an MS patient means they are at higher risk of getting infected.

Moreover, many studies have been conducted among the general public in Saudi Arabia, assessing their knowledge, attitude, and behavioral practice toward the COVID-19 pandemic. These studies have reported an adequate understanding regarding the infection spreads, how to protect themselves, as well as which age categories are most prone to be affected [26]. Another study showed that more than 81% of surveyed Saudis had satisfying knowledge regarding COVID-19 infection [23]. Furthermore, a significant positive association has been found between Saudis’ awareness, attitude, and behaviors, suggesting a higher degree of awareness and better attitude and behaviors [27]. Data also showed that 17% of patients attended a social event involving many people. Also, 28.0% of the patients reported being in crowded places. Several worldwide publications have highlighted the potential role of KAP studies on MS patients; a study conducted in Iran presented that most respondents believed closing crowded places increase the infection risk, and 97% thought border closure is helpful [28]. In Montenegro, men revealed that they intensified their safety behavior (handwashing, disinfection) and expressed that isolation measures had a significant negative impact on them compared to female patients, which suggested that men are more in danger of becoming infected [29]. The primary limitation of our study is that data were collected online by a self-reported survey which might be influenced by reporting bias. Furthermore, the study has not covered all MS patients from all regions of Saudi Arabia, which might affect the generalizability of our conclusion. Additionally, respondents have different responses based on their location at the time of response within Saudi Arabia, given that the incidence of cases and the wave of new cases might affect them differently.

## Conclusions

MS patients’ risk of COVID-19 might be linked to their knowledge, attitude, and behaviors. Our results suggest that although MS patients have a high knowledge and good attitude and behaviors, it is still essential to have health education programs among MS patients to reduce the risk of SARS-CoV-2 infection and the impact of the COVID-19 pandemic on MS patients’ care. Knowledge, attitude, and behaviors toward the COVID-19 pandemic can highlight the importance of education programs and translate the findings into action to minimize the disease risk.
